# Automated Processing and Deviation Analysis of 3D Pipeline Point Clouds Based on Geometric Features

**DOI:** 10.3390/jimaging12030115

**Published:** 2026-03-09

**Authors:** Shaofeng Jin, Kangrui Fu, Chengzhen Yang, Huanhuan Rui

**Affiliations:** College of Mechanical and Electrical Engineering, Nanjing University of Aeronautics and Astronautics, Nanjing 210016, China; fukr_nuaa@nuaa.edu.cn (K.F.); yangchengzhen@nuaa.edu.cn (C.Y.); ruihuanhuan@nuaa.edu.cn (H.R.)

**Keywords:** 3D laser scanning, point cloud processing, 3D measurement, industrial software

## Abstract

To meet the strict non-contact measurement requirements for the assembly of aircraft engine pipelines and to overcome the limitations of the traditional three-dimensional laser scanning workflow, this study proposes an automated pipeline point cloud processing and deviation analysis framework. Through a standardized three-dimensional laser scanning procedure, high-resolution pipeline point clouds are obtained and preprocessed. Based on the geometric characteristics of the pipeline, automated algorithms for point cloud feature segmentation, axis extraction, and model registration are developed. Particularly, the three-dimensional extended Douglas–Peucker (DP) algorithm is introduced to achieve efficient point cloud downsampling while retaining necessary geometric and structural features. These algorithms are fully integrated into a unified software platform, supporting one-click operation, and can automatically analyze and obtain five key types of pipeline deviations: angular deviation, radial deviation, axial deviation, roundness error, and diameter error. The platform also provides intuitive visualization effects and comprehensive report generation functions to facilitate quantitative inspection and analysis. Test results show that the proposed method significantly improves the processing efficiency and measurement reliability of complex pipeline systems. The developed framework provides a powerful practical solution for the automated geometric inspection of aircraft engine pipelines and lays a solid foundation for subsequent quality assessment tasks.

## 1. Introduction

Pipelines are external components that ensure the reliable operation of aero-engines. At present, sealing performance after assembly often fails to meet design requirements, and such issues are typically resolved through repeated disassembly and local rework, which severely degrades assembly efficiency. If a more efficient pipeline measurement scheme could be developed to accurately capture geometric deviations and provide data support for subsequent sealing performance prediction and pipeline reshaping, overall assembly efficiency could be significantly improved. Considering the structural characteristics of pipelines, machine vision technology is introduced to achieve high-efficiency, non-contact measurement. Compared with the general industrial measurement requirements, the piping of an aircraft engine has the characteristics of a dense spatial layout, significant obstruction effects caused by adjacent components, and reflective surfaces of the metal material. These factors pose numerous challenges to 3D laser scanning, including incomplete data collection, multiple path reflections, and background interference caused by fixed devices and the engine casing [1]. Extensive research has been conducted on how to efficiently and accurately process point cloud data obtained from scanning into meaningful information that describes position, shape, and dimensional characteristics. Existing point cloud processing methods can generally be classified into three categories: traditional statistical- and geometry-based approaches, deep learning–based black-box methods, and solid geometry-oriented parametric modeling methods tailored for engineering structures.

The first category comprises traditional statistical-based point cloud processing methods. These approaches mainly rely on spatial relationships among points, local neighborhood statistical features, and variations in normal vectors to perform noise removal, down sampling, feature extraction, and reverse modeling. Such methods offer advantages including high computational efficiency and strong interpretability, making them well suited for scenarios with heavy noise and relatively regular geometric structures.

In terms of fitting and denoising, Luo [2] proposed the VWC-LSPF method, which combines voxel-weighted centroids with least-squares plane fitting to achieve robust parameter estimation under high-noise conditions through iterative updates. Li [3] developed an engineering noise filtering algorithm based on local statistical features and adaptive thresholds, effectively suppressing unstructured noise while preserving edge geometry. Xi [4] modeled scanning errors and established a distribution estimation framework for point cloud noise in engineering environments, providing a theoretical foundation for subsequent accuracy control and denoising. For point cloud simplification, Chao [5] proposed a framework that jointly analyzes point density and local geometric features, improving boundary extraction accuracy through a geometry-weighted strategy. Yang [6] constructed feature-preserving simplification rules based on local differential statistics, enabling structural consistency to be maintained even under high compression ratios. In registration tasks, Wang [7] enhanced robustness through multi-constraint ICP and adaptive feature matching strategies, which are applicable to multi-view and heavily occluded engineering environments. For feature recognition and error modeling, Ma [8] improved boundary extraction accuracy by analyzing neighborhood point tensors, providing reliable data support for subsequent geometric modeling. Verhoeven [9] proposed a method for shape fitting based on Mahalanobis distance after considering the uncertainty of different sizes and directions of each data point. Lipschütz [10] investigated point cloud error propagation and established error-constrained models, offering theoretical guidance for precision optimization in point cloud fusion and stitching. Yan [11] and Zheng [12] further proposed feature-oriented boundary recognition strategies, demonstrating significant advantages in complex surfaces and scenarios with pronounced local geometric variations.

Overall, traditional statistical-based methods are characterized by simple computational procedures, strong interpretability, and stable results, making them particularly suitable for engineering scenarios with simple structures and high efficiency requirements. However, their capabilities are limited when dealing with complex structural analysis and advanced feature recognition in point cloud data.

The second category consists of deep learning-based point cloud processing methods, including neural network architectures such as PointNet, DGCNN, and Transformer-based models. With sufficient training data, deep learning approaches have demonstrated outstanding performance in tasks such as point cloud segmentation, feature recognition, and point cloud completion, thus becoming a major research direction in recent years.

In the area of completion and generation, Zhan [13] systematically classified point cloud completion techniques and reviewed the evolution of CNNs, graph neural networks, Transformers, and diffusion models in completion pipelines. Tang [14] proposed a multi-stage point cloud upsampling method that achieves stable structural completion through guided feature fusion. Ding [15] employed hierarchical diffusion models to generate point clouds with global consistency, enhancing adaptability to complex shapes. Yang [16] further introduced a multimodal conditional diffusion approach that enables controllable completion using external information. For semantic segmentation and structural parsing, Yu [17] proposed PipeSegNet for pipeline scenarios, achieving high-precision recognition and segmentation of complex pipeline components by incorporating feature enhancement and structural priors. Wang [18] and Si [19] leveraged Transformers and graph neural networks to capture long-range dependencies, improving structural understanding of large-scale point clouds. Song [20] introduced a local-structure-aware module that enhances local feature representation through self-attention mechanisms. Kumari [21] proposed a coarse-to-fine two-stage completion network that maintains global geometric consistency even under severe data loss. Sun [22] put forward a hierarchical motion estimation/motion compensation (ME/MC) framework and built a dual-attention-based KNN network, significantly improving the compression efficiency of point cloud data. In terms of engineering deployment, Tian [23] proposed a cross-domain framework combining self-supervised learning and domain adaptation modules, mitigating distribution shifts caused by different measurement devices through invariance constraints. Lian [24] developed a perception network tailored for low-density point clouds, enabling stable semantic representations in sparse scenarios. Zhao [25] introduced a multi-task network integrating denoising, enhancement, and segmentation, improving robustness in real-world environments. Deng [26] enhanced structural representation through shape-aware feature encoding and adaptive normalization, thereby improving overall point cloud parsing accuracy.

In summary, deep learning approaches address high-dimensional semantic tasks that are difficult for traditional methods, offering irreplaceable advantages in complex structural understanding, completion prediction, and cross-domain adaptation. However, the trained models generally suffer from limited interpretability, strong data dependence, and sensitivity to noise, which may pose challenges for precise dimensional measurement of engineering components.

The third category is based on solid geometry theories and algorithms. By incorporating the geometric characteristics of the measured object, these methods extract key geometric features from point cloud data and transform them into parametric models. The primary objective of reverse engineering is to map point cloud data, through a series of processing steps, into editable, computable, and engineering-meaningful CAD-based parametric as-built models. Reconstruction methods grounded in solid geometry and parametric modeling therefore represent a critical direction for engineering applications.

In digital twin and engineering modeling, Xie [27] comprehensively reviewed the parametric mapping mechanisms from point clouds to 3D models, with particular emphasis on downsampling as a key technical bottleneck, and summarized a wide range of point cloud sampling strategies. Li [28] proposed a digital twin method for large-scale pipeline systems based on geometric constraints between point clouds and 3D models, achieving structured representation and high-precision reconstruction of complex pipelines. Lu [29] and Luo [30] introduced incremental component extraction and parameter optimization strategies, enabling stable fitting of engineering components under occlusion and noise through iterative geometric updates. Ding [31] proposed a clearance detection method for aero-engine pipelines based on centerline geometric criteria, enabling automatic quantification of critical clearances in complex structures. Zhang [32] utilized geometric priors of linear structures to achieve robust axis fitting for pipes, beams, and columns. In deviation detection and parametric modeling, Liu [33] developed a parametric representation model for curved surfaces, improving registration accuracy between point clouds and models. Zhu [34] constructed lightweight point cloud inspection models using command-generation methods, facilitating point cloud processing in engineering field applications. Li [35] proposed a structural deformation field modeling approach that infers structural deformation from point clouds and maps it to parametric geometry, enabling component-level geometric deviation analysis. Feng [36] employed geometric consistency constraints to achieve spatial registration between point clouds and multi-component 3D models, supporting assembly verification and deviation detection. Li [37] and Zeng [38] further extended this line of work by enhancing parametric reconstruction completeness from the perspectives of component contour modeling and feature constraints, respectively.

Overall, a point cloud-to-3D model comparison based on as-built measurement data is more suitable for component inspection tasks, while parametric modeling methods provide high-precision geometric representations and are therefore the most appropriate point cloud processing techniques for industrial metrology.

Each of the three categories excels at addressing different aspects of point cloud analysis. Traditional statistical methods provide geometric foundations and noise control, deep learning enhances semantic understanding and structural reasoning, and parametric modeling ensures the precision of analysis results. However, existing studies have not yet achieved deep integration between point cloud data and parametric models. There remains a need to establish an automated analysis pipeline from point clouds to parameters, as well as to develop industry-oriented, customized point cloud analysis algorithms. Building upon the aforementioned techniques, further research is required to form a comprehensive pipeline deviation analysis methodology based on scanned as-built point cloud data.

## 2. Pipeline Point Cloud Data Acquisition and Preprocessing

The pipeline systems of aero-engines are characterized by a wide variety and complex geometries. To address the substantial demand for pre-assembly inspection of metallic pipelines on-site, a high-efficiency and highly compatible 3D laser scanning technology was adopted. Accordingly, 3D laser scanning equipment was strategically deployed at the assembly site. To ensure the quality of the acquired point cloud data and enhance measurement accuracy, standardized procedures for equipment registration and scanning protocols were established.

Following the acquisition of the initial point cloud data through 3D laser scanning, the presence of outliers and voids was observed, primarily due to surface reflectivity of the pipelines and ambient lighting interference in the measurement environment. To mitigate these issues and improve the integrity of the point cloud, a series of error elimination and preliminary data repair processes were implemented.

To guarantee high measurement accuracy, the point cloud was initially captured at an extremely high spatial resolution. However, considering the computational burden associated with high-density data during subsequent analysis, a down sampling process was applied to the point cloud to optimize computational efficiency without significantly compromising data fidelity.

### 2.1. Measurement Site Setup and Standardized Measurement Procedure

Based on preliminary research and experimental validation, the following conclusions have been drawn: traditional contact-based measurement methods exhibit inherent limitations in measurement efficiency and are inadequate for handling the complex geometries of spatial pipeline components. Although conventional photogrammetry techniques are simple to operate and offer reliable accuracy, they require labor-intensive setup procedures and often fail to provide sufficient data density for reconstructing pipeline geometries with high fidelity. In contrast, 3D laser scanning technology offers significant advantages, including non-contact operation, high measurement efficiency, superior accuracy, and strong reliability. Furthermore, the resulting point cloud data are characterized by high versatility and rich detail, enabling the accurate representation of both individual pipe segments and complex assembled pipeline structures. Consequently, 3D laser scanning systems were selected as the primary data acquisition method for this study.

**Figure 1:**
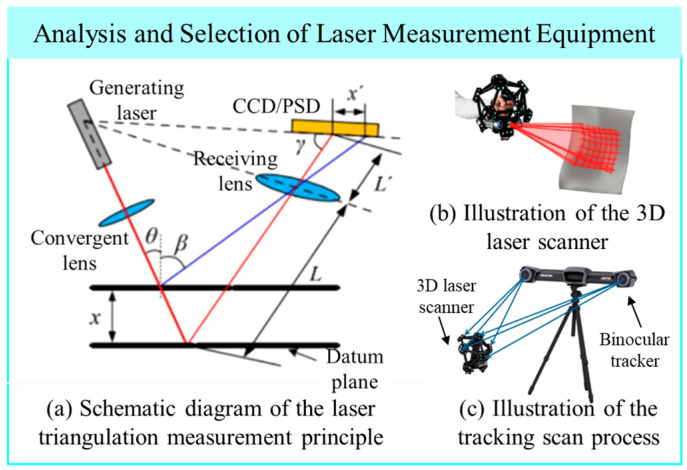
Illustration of 3D laser scanning principle and equipment selection. (**a**) Schematic diagram of the laser triangulation measurement principle; (**b**) Illustration of the 3D laser scanner; (**c**) Illustration of the tracking scan process.

3D laser scanning is an emerging measurement technology that has gained widespread adoption in recent years, particularly in the field of reverse engineering. Distinguished from laser scanning systems used in the architecture and civil engineering industries, the measurement requirements in aerospace especially in the assembly of aero-engine pipelines demand higher precision over smaller spatial ranges. In such contexts, laser triangulation-based measurement systems are particularly well-suited. The core principle involves emitting a laser beam from the scanner toward the target object, receiving the reflected signal at the opposite end of a fixed baseline, and recording the angle between the incident and reflected beams. With the known baseline length and the measured angles, the distance between the instrument and the target point can be accurately calculated based on triangulation. This geometric relationship formed by the laser emitter, the target point, and the receiver constitutes the foundation of the triangulation-based laser measurement method, from which the technique derives its name. The measurement principle is illustrated schematically in Figure 1a.

As shown in Figure 1b, the operating status of the laser scanner based on the triangulation principle is constrained by the limited scanning field of view. Consequently, multiple scanning passes are often required and must be registered to obtain a complete point cloud of the target object. Traditional multi-view registration typically relies on the application of artificial features—such as speckle patterns or reflective targets on the surface of the measured object to serve as reference markers during data stitching. However, due to the harsh service environment of aero-engine pipelines, applying such features via surface spraying or adhesive targets is not feasible.

To overcome the limitations of conventional laser scanning systems in the pipeline assembly process, a tracking-type 3D laser scanning system was adopted. This system employs a C-Track dual-camera unit that simultaneously captures spatial positioning markers and utilizes software algorithms based on triangulation to determine the pose of the scanning probe and its surrounding environment. This enables marker less point cloud registration, as illustrated in Figure 1c.

**Figure 2:**
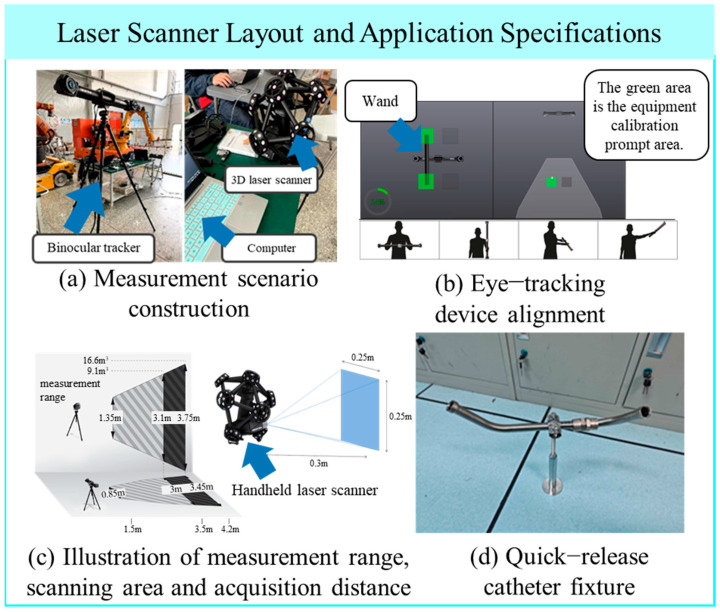
Specification for Layout and Usage Procedures of 3D Laser Scanning Equipment. (**a**) Measurement scenario construction; (**b**) Eye-tracking device alignment; (**c**) Illustration of measurement range, scanning area and acquisition distance; (**d**) Quick-release catheter fixture.

**Figure 3:**
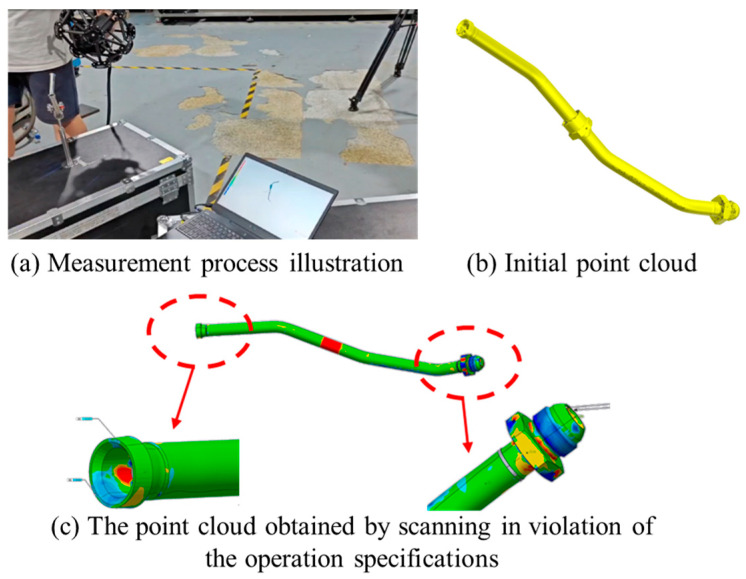
Illustration of the Scanning Process and the Initial Point Cloud. (**a**) Measurement process illustration; (**b**) Initial point cloud; (**c**) The point cloud obtained by scanning in violation of the operation specifications.

The scene layout of the three-dimensional laser scanning equipment is shown in Figure 2a. Upon determining the appropriate measurement mode, a series of preparatory steps must be completed to ensure the smooth execution of the scanning process and the reliability of the acquired data. These steps include equipment calibration, measurement platform setup, measurement feature arrangement, and object optimization. The specific procedures and associated requirements are as follows:

(1) Laser Scanner Calibration: Place the calibration board on a stable and level surface. Adjust its orientation relative to the C-Track unit. Activate the scanner’s acquisition mode and move the scanner to predefined positions indicated in the viewer interface. Complete the calibration at multiple positions as guided.
(2) Stereo Tracker Calibration: Use a calibration wand to perform full spatial calibration of the stereo tracking system. To enhance tracking accuracy during markerless scanning, the wand is moved by the operator in horizontal, vertical, and two depth directions, enabling comprehensive 3D calibration. The process is depicted in Figure 2b.
(3) Defining the Operational Workspace: After calibration, the effective working volume of the stereo tracker is determined. The handheld laser scanner must remain within this region to ensure point cloud accuracy. The scanner’s field of view and optimal acquisition distance are shown in Figure 2c. A suitable scanning setup is then constructed based on the geometry of the target pipeline. This includes the use of quick-release fixtures that allow the pipeline to be repositioned from various angles to capture a more complete point cloud. A sample fixture for holding the pipeline during scanning is shown in Figure 2d.
(4) Environmental Optimization: In cases where the scanning environment is poorly lit or the pipeline surface exhibits strong reflectivity that compromises data acquisition, supplemental lighting sources should be added. Additionally, non-invasive treatments—such as applying masking tape or matte coatings—can be used to improve data quality.
(5) Pipeline Scanning Strategy: A scanning strategy tailored to the structural characteristics of the pipeline is established. First, a coarse scan is conducted to obtain a rough spatial representation and the overall shape of the component. Then, detailed scans are performed on critical features such as bends and joints. This involves hovering the scanner near key regions and executing small, deliberate oscillations to enhance local point density and capture critical feature geometries with higher precision. Finally, the scanning path should be planned to minimize the inclusion of background surfaces or fixtures, which may introduce unwanted outliers and noise into the point cloud. The actual measurement process is shown in Figure 3a.

When the scan was carried out formally, the point cloud sampling density set was 0.03 mm. Moreover, during scanning, it is recommended to maintain the scanning head perpendicular to the surface of the pipeline to maximize measurement efficiency. In cases where detailed scanning of local features is required, a slight tilt of the scanner is acceptable to trade efficiency for improved completeness of point cloud data. An example of the resulting raw point cloud is presented in Figure 3b.

The scanning process should strictly follow the operation procedures. If it is not carried out as required, it may result in point cloud data with excessive local deviations. During the subsequent analysis, this will introduce significant errors, as shown in Figure 3c.

### 2.2. Initial Noise Removal from Raw Point Cloud Data

After obtaining the point cloud data through scanning, the first step is to perform noise data processing to eliminate the noise points generated during the scanning process. During 3D laser scanning of pipeline components, the resulting point cloud data inevitably contain noise, which can significantly interfere with subsequent data processing and analysis. Therefore, a preprocessing step is essential prior to any analytical procedures in order to remove such noise. Noise in point cloud data can generally be classified into two distinct categories. The first type consists of external outliers—points that lie outside the actual geometry of the target pipeline. These are typically introduced when non-target objects are inadvertently scanned during the measurement process or due to reflective surfaces causing erroneous returns. The second type refers to internal noise, which arises from systematic and random errors within the measurement system itself. These noise points are embedded within the pipeline point cloud and can distort the true geometry if not properly addressed.

Given the differing characteristics and origins of these two types of noise, distinct denoising strategies must be employed to handle each case effectively.

For outliers caused by environmental factors, such as material reflectivity or lighting conditions—leading to the presence of scattered noise near the pipeline surface (including unintended point clouds from tables, fixtures, or other objects), a clustering-based approach using a KD-tree structure is particularly effective. Clustering algorithms classify the point cloud by computing the distance between each point and its neighboring points within a defined neighborhood. Because such outliers are typically sparse and constitute a small proportion of the overall dataset, their average distance to surrounding points tends to exceed that of the actual pipeline data. If a point’s mean distance to its neighbors surpasses a predefined threshold, it can be classified as an outlier and subsequently removed. This process can be iteratively applied to eliminate the majority of isolated noise points.

For noise induced by system errors and random fluctuations—especially when such noise exhibits statistical regularity—Gaussian filtering is a commonly used technique. Gaussian filtering is a linear smoothing method that performs a weighted average of neighboring data points within a fixed range. It effectively reduces noise while preserving essential geometric features of the point cloud.

**Figure 4:**
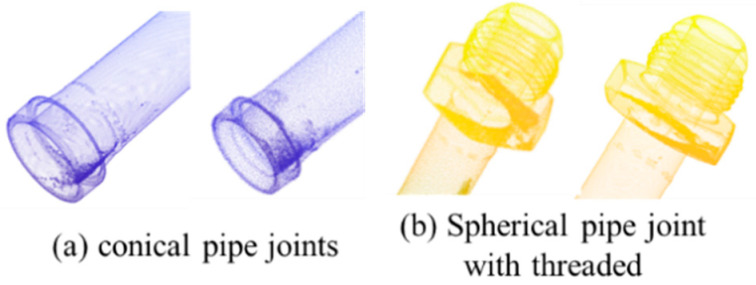
Comparison Figure of Point Cloud Denoising Effect. (**a**) conical pipe joints; (**b**) Spherical pipe joint with threaded.

By applying the aforementioned denoising methods, the point cloud becomes smoother and more continuous while maintaining the fine geometric details of the pipeline structure. This denoised dataset provides a solid foundation for subsequent analysis and feature extraction. A comparison of local pipeline features before and after denoising is shown in Figure 4a,b.

## 3. Point Cloud Data Analysis with Considering of Pipeline Geometric Features

**Figure 5:**
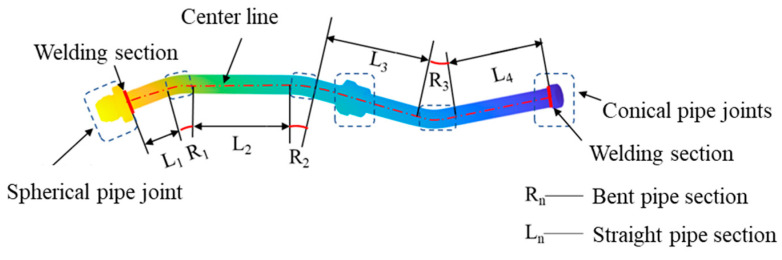
Illustration of Pipeline Feature Split.

The pipeline of aerospace engine is assembled by welding together pipes and joint components. The pipe section is typically formed by bending straight pipes, resulting in a centerline composed of alternating linear and circular arc segments. Accordingly, the duct geometry can be segmented into straight and curved sections. Given the uniform diameter of the pipe, its spatial shape can be effectively represented using a combination of the centerline path and a constant radius, as illustrated in Figure 5. This geometric characteristic enables efficient and accurate analysis and parameterization of the duct’s point cloud data by extracting and leveraging centerline-based features.

### 3.1. Pipeline Point Cloud Segmentation

Point cloud segmentation aims to obtain straight segments of pipelines to extract key information such as the axis line. To achieve efficient and accurate point cloud segmentation, the characteristics of four point cloud segmentation algorithms were compared. Since this study has regular structural features corresponding to the segmentation task and certain requirements for computational efficiency, the SDF method is more applicable. The comparison of methods and applicable scenarios are shown in Table 1.

The Shape Diameter Function (SDF) is an effective descriptor for local diameter features of three-dimensional models, capable of reflecting the local geometric size information at each sampled point within point clouds or mesh models. Traditionally, SDF has been applied primarily to 3D mesh models, where it intuitively characterizes local shapes by computing the average diameter inside the model at vertices or facets. For aero-engine pipeline point cloud data, which exhibit significant curvature variations, SDF effectively differentiates structural regions such as straight segments, bends, and pipe joints.

However, the sparsity and lack of topological structure in point cloud data pose challenges for direct application of conventional SDF methods. To address these characteristics, this work proposes an adaptive modification to the SDF computation for point clouds. Specifically, a set of *n* key points are uniformly sampled from the point cloud as calculation bases. For each sampled point, a conical region with an apex angle of 120° is constructed along the inverse direction of its normal vector, serving as the central axis. Points falling within this cone are selected, and the lengths of the rays connecting these points to the sampled point are computed. A cosine-weighted scheme based on the angle between each ray and the central axis is applied to weigh these lengths, and the weighted shortest ray length is taken as the SDF value for that sampled point. This approach balances the effects of local point cloud sparsity and data loss caused by measurement occlusions, ensuring accuracy and robustness of the extracted SDF features. The statistical distribution of SDF values within each segmented block serves as an effective descriptor of the local shape, providing a sound basis for subsequent segmentation.

After the initial region partitioning based on SDF, fragmentation and discontinuity issues are addressed through an optimization process incorporating the mutual visibility principle and region growing algorithm. The mutual visibility principle asserts that any two points within the same region should have an unobstructed line of sight, thus guaranteeing spatial connectivity and geometric consistency. Starting from seed points, the region growing algorithm iteratively aggregates neighboring points that satisfy both mutual visibility and SDF similarity criteria, expanding regions until convergence. Additionally, cross-region visibility checks are performed on boundary points of adjacent fragmented segments to determine and merge segments when appropriate, effectively mitigating over-segmentation. This strategy significantly enhances the completeness and accuracy of the segmentation results.

**Figure 6:**
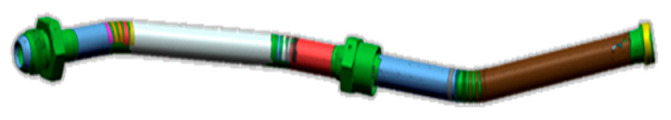
Pipeline point cloud segmentation result display.

In summary, the proposed point cloud segmentation method based on the Shape Diameter Function and mutual visibility principle achieves structured partitioning of aero-engine pipeline point clouds, accurately distinguishing straight segments, bends, and pipe joints, as illustrated in Figure 6. The method not only improves segmentation precision and continuity but also establishes a solid foundation for subsequent geometric modeling, assembly inspection, and performance evaluation.

### 3.2. An Improved DP Algorithm for 3D Point Cloud Simplification

After segmenting the pipeline point cloud surfaces, down sampling is often required to reduce the number of points and improve computational efficiency in geometric feature extraction. This paper proposes an improved three-dimensional Douglas–Peucker (DP) algorithm tailored for point cloud down sampling. Building upon the classical DP algorithm, the proposed method introduces a neighborhood-based evaluation mechanism that leverages geometric features of adjacent triangular facets. By calculating the shortest distance from a candidate point to the local triangular facets within its neighborhood, the algorithm more accurately assesses the significance of each point.

Experimental results demonstrate that the proposed method effectively reduces point cloud data size while preserving key geometric features of the 3D model. This approach is applicable to large-scale point cloud processing in fields such as 3D printing, computer graphics, and geographic information systems.

The DP algorithm, originally designed for trajectory simplification, employs a geometric criterion based on point-to-line distances. Under the constraint that the overall shape remains approximately unchanged, it recursively removes redundant points. In the 2D case, the DP algorithm constructs a line segment between the start and end points of a curve, computes the perpendicular distance of all intermediate points to this line, and compares the maximum distance *d* to a preset threshold D. If *d* ≤ D, the curve segment is approximated by the line segment, and intermediate points are discarded. Otherwise, the point with the maximum distance is retained, and the curve is recursively subdivided at this point until all retained points satisfy the distance criterion. The DP algorithm has been applied in scenarios such as trajectory sparsification for ships [39] and trajectory planning and simplification in radiation fields [40]. Some researchers have also utilized this algorithm in the processing of boundary points of point cloud data [41]. Based on references from other literature, this paper is the first to extend the DP algorithm to the three-dimensional domain.

Unlike the classical DP algorithm that only addresses 2D curves and uses point-to-line distances as the simplification criterion, the present work extends the concept to 3D surface point clouds. This is achieved by constructing a local reference plane within the surface and evaluating the geometric consistency of points relative to this plane, enabling effective simplification of pipeline surface point clouds.

Specifically, an initial candidate point set is selected from the surface point cloud, from which a point *P* is chosen for evaluation. Using *P* as the center, a local reference plane is constructed by fitting a plane through its three nearest neighbors on the surface, approximating the local geometric shape in the region. The orthogonal distance *d* from *P* to this reference plane is computed and compared against a preset geometric consistency threshold D. If *d* > D, the point *P* is considered geometrically significant and retained. Conversely, if *d* ≤ D, *P* is regarded as redundant within the surface and removed. This evaluation process iterates over the point cloud until all points have been processed, with iterations controlled by checking whether all points meet the retention criterion. After completion, the downsampled point cloud segments are merged according to their original geometric positions, producing a final point cloud that satisfies geometric accuracy requirements.

**Figure 7:**
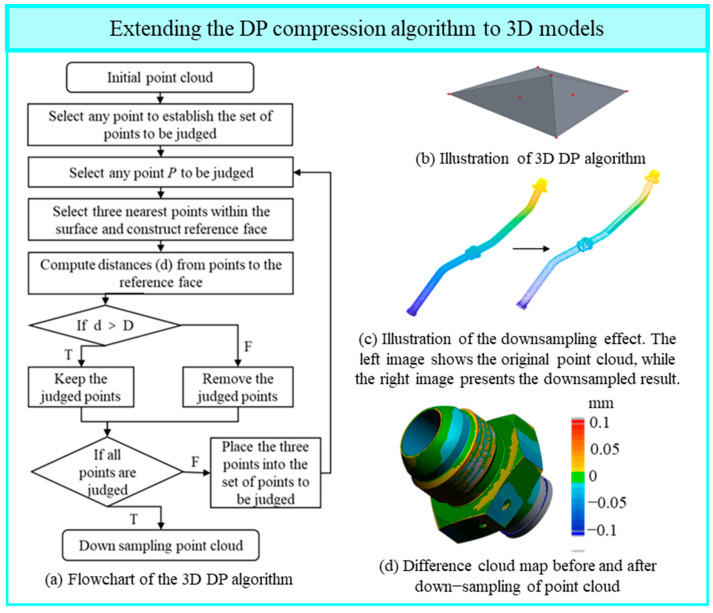
3D DP algorithm: improvement principles and result visualization. (**a**) Flowchart of the 3D DP algorithm; (**b**) Illustration of 3D DP algorithm; (**c**) Illustration of the downsampling effect. The left image shows the original point cloud, while the right image presents the downsampled result; (**d**) Difference cloud map before and after down-sampling of point cloud.

A flowchart illustrating the algorithm is shown in Figure 7a, and a geometric schematic of the computation process is presented in Figure 7b. After expanding and improving the DP algorithm to three dimensions, the specific threshold D needs to be adjusted based on the sampling results of the point cloud data. This is because when conducting error analysis on the point cloud obtained from scanning, the acceptable error is 0.1 mm. Therefore, the precision loss should be controlled within 0.1 mm. Based on this, multiple tests were carried out, and it was found that when D is set to 0.08 mm, the maximum deviation between the point clouds before and after processing still does not exceed 0.1 mm. At this point, point cloud data reduction sampling was achieved while retaining as many features as possible. This ensures that the point cloud sampling process does not cause severe shape changes and precision loss, and also minimizes the number of points and improves the computational efficiency. The effect of point cloud data reduction sampling is shown in Figure 7c, and the cloud graph of the difference between the two groups of point clouds before and after reduction is shown in Figure 7d.

By extending and improving the DP algorithm for 3D applications, the proposed method achieves effective point cloud down sampling while preserving critical geometric features. This ensures minimal shape distortion and precision loss during simplification, while significantly reducing point counts and improving computational efficiency. The threshold D should be adjusted based on the specific sampling characteristics of the point cloud data.

### 3.3. Pipeline Centerline Feature Extraction Based on Elliptical Fitting

**Figure 8:**
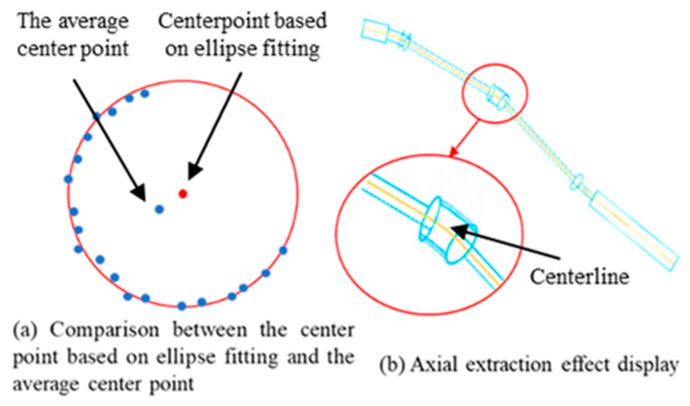
Principle and results of pipeline centerline extraction. (**a**) Comparison between the center point based on ellipse fitting and the average center point; (**b**) Axial extraction effect display.

The centerline of a pipeline serves as a fundamental structural feature of the pipeline model, accurately representing the pipeline’s curve trajectory and geometric shape. It is widely used in pipeline matching, recognition, and reverse engineering. For large-scale aerospace engine pipeline systems characterized by uneven point distribution and complex structures, traditional centerline extraction algorithms often struggle to yield satisfactory results. This issue primarily arises from the incomplete point cloud data caused by measurement constraints such as limited sensor viewpoints and structural occlusions. Consequently, the centerline computed by averaging cross-sectional points may deviate from the true geometric centerline. Typically, the computed local L1 median tends to be biased toward the side with more complete point data, thus compromising the accuracy and representativeness of the extracted centerline, as illustrated in Figure 8a.

To address this problem, this paper proposes an ellipse fitting–based centerline centralization method to precisely adjust each extracted pipeline centerline, ensuring closer alignment with the true pipeline center position. The implementation proceeds as follows: For each pipeline centerline, excluding the endpoints, a cross-sectional plane is constructed at each intermediate centerline point. This cross-sectional plane is centered at the point and oriented with a normal vector perpendicular to the vector formed between the current centerline point and its successor, thereby guaranteeing that the plane is locally orthogonal to the pipeline’s axial direction.

Next, points in the input point set P within the neighborhood of the centerline point are projected onto this cross-sectional plane, yielding a two-dimensional cross-sectional point set near the centerline point. The optimal fitting ellipse was calculated using the least squares elliptical fitting algorithm, The ellipse center represents the local pipeline center at this cross-section. The centerline point is geometrically centralized by shifting it along the plane to coincide with the fitted ellipse center.

After all centerline points have been centralized in this manner, a smoothing operation is performed along the entire centerline to eliminate possible local fluctuations introduced during adjustment, ensuring continuity and smoothness of the centerline. This centralization process effectively corrects deviations caused by point cloud data loss, enhancing the precision and reliability of pipeline centerline extraction. It provides more accurate foundational data for subsequent pipeline geometric analysis and reverse modeling. The final extracted pipeline centerline is shown in Figure 8b.

## 4. Pipeline Point Cloud Data Processing Software Development and Functional Testing

Based on the PreSys 3D geometry software (Software version 2025R2.) platform, a dedicated plugin for pipeline point cloud data processing has been developed to automate the registration of measured point clouds with theoretical models and the computation of deviations. The primary focus is on the application modules of the software, whose main functionalities are as follows:

(1) Model and Point Cloud Import and Preprocessing: Correctly import pipeline geometry model files (.prt) and point cloud files (.stl), and accurately display them within the software interface. Automatically extract features from the theoretical model, and perform filtering, denoising, and feature-based segmentation on the point cloud data.
(2) Registration of the model and point cloud: The ICP (Iterative Closest Point) algorithm is used for the registration of the model and the point cloud. The registration matrix is calculated and the registration process is automated.
(3) Feature Extraction and Deviation Analysis: Extract the centerlines of both the theoretical pipeline model and the point cloud model. Along the centerline direction, extract the boundary surfaces at both ends of the pipeline model, analyze their positions and normal vectors, and automatically calculate feature deviations. The deviation data are output in the form of comprehensive reports.

### 4.1. Model Import and Automatic Preprocessing

**Figure 9:**
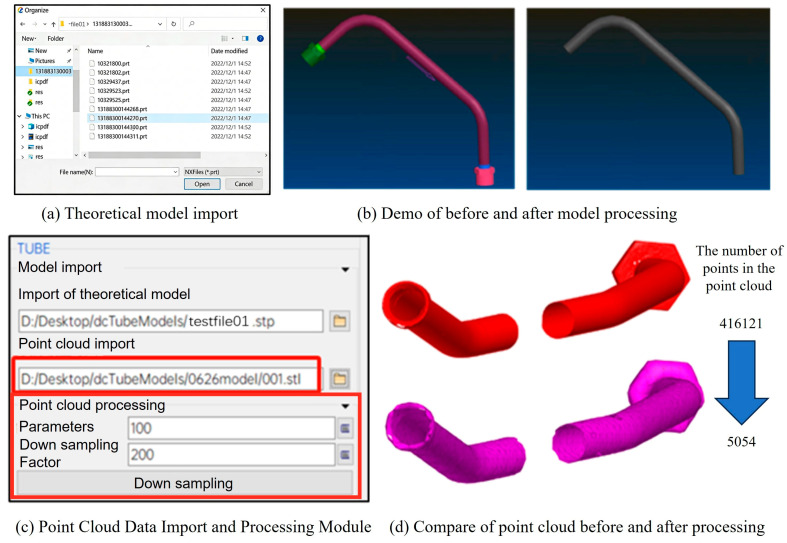
Model import and automatic preprocessing module. (**a**) Theoretical model import interface; (**b**) Demo of before and after model processing; (**c**) Point Cloud Data Import and Processing Module; (**d**) Compar of point cloud before and after processing.

In the current design, manufacturing, and assembly processes of aero-engine pipelines, the theoretical pipeline models are primarily created using UGNX software (Unigraphics NX is an engineering software developed by Siemens PLM Software. It enables the construction and analysis of various complex entities and is often used as a modeling software in the aerospace industry. Software version NX 2406.9100.), with the main file format being .prt. Therefore, targeted optimization for this format is necessary. We have analyzed the encoding rules of this format and implemented the corresponding model import functionality. The interface for opening theoretical model files is shown in Figure 9a.

After importing the theoretical model, there are some redundant pieces of information, including the arrows indicating the flow direction and the nuts pre-installed on the catheter, which do not participate in the current deviation analysis. Manually preprocessing each model is highly inefficient; hence, we developed an automatic preprocessing function for the theoretical model. The effect of this model processing is illustrated in Figure 9b.

To ensure the quality of scanned data, we selected .stl files to export point cloud data. STL represents a triangular facet model, which preserves the relative positions of scanned points while retaining key directional features of internal and external surfaces. Accordingly, we developed and optimized the STL file reading module. The point cloud data import and processing module is shown in Figure 9c. By inputting simplification parameters and clicking the “Simplify” button, the previously described point cloud segmentation and down sampling algorithms are automatically invoked, enabling one-click simplification of the point cloud model, thus improving the accuracy of subsequent feature analysis.

To address common errors or issues that may affect simulation results, the model inspection menu provides tools to evaluate model quality. Default parameters for each inspection criterion can be configured by users through commands in the Tools/Options center. For instance, detected duplicate nodes can be merged, and duplicate elements can be assigned to a separate part layer for independent processing. The inspection functions include checking and deleting duplicate nodes, checking and deleting duplicate elements, and automatic completion of missing nodes. The effect comparison before and after point cloud data processing is illustrated in Figure 9d.

### 4.2. Automatic Point Cloud and Model Registration

Registration primarily refers to transforming the measured point cloud data and the theoretical model point cloud into a common coordinate system, maximizing their overlap to facilitate subsequent feature analysis and deviation calculation. The most widely used point cloud registration algorithm is the Iterative Closest Point (ICP) algorithm. Its fundamental principle is to iteratively perform two steps to achieve registration: first, for each point in the source point cloud, find the closest corresponding point in the target point cloud; second, compute the optimal rigid transformation (including rotation and translation) that minimizes the distance errors between corresponding point pairs. This process repeats until the error converges or a preset iteration limit is reached, thereby achieving accurate alignment of the two point clouds. Since ICP is a standard algorithm and not the focus of innovation in this work, detailed descriptions are omitted here.

**Figure 10:**
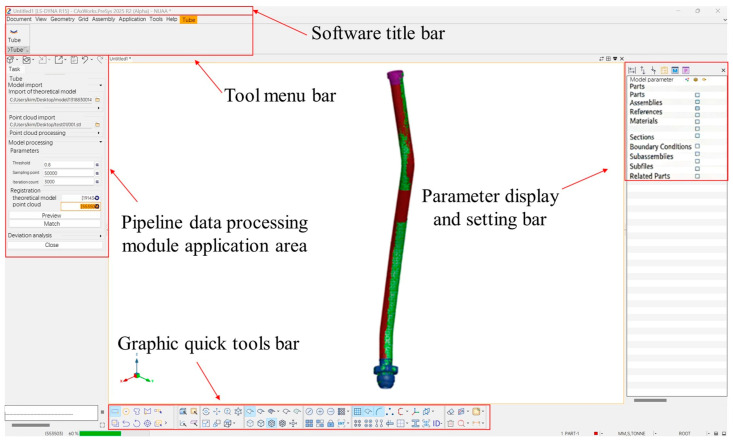
Registration module display.

In the pipeline registration interface, users first select the theoretical pipeline model and the point cloud model. The program provides a set of default registration parameters, which users may adjust as needed. By clicking the “Register” button, the theoretical model is transformed according to the computed registration result. The registration process supports multiple iterations and manual adjustments of the model position. The registration result between the actual point cloud model and the theoretical point cloud model is shown in Figure 10.

### 4.3. Point Cloud Feature Extraction and Deviation Solving

The deviations calculated primarily include five types: angular deviation, radial deviation, axial deviation, roundness error, and diameter error. Angular deviation refers to the angle between normal vectors. Radial deviation is the projection of the distance between centroids onto the circular plane. Axial deviation is the projection of the centroid distance along the normal vector. Roundness error is defined as the difference between the maximum and minimum radii of an ellipse. Diameter error is the difference between the average radius of the ellipse and the radius of the ideal circle. The above five deviations are all calculated based on point cloud data. The accuracy of the point cloud data is 0.1 mm. The accuracies of the four linear dimension types of errors, radial deviation, axial deviation, roundness error and diameter error, are all 0.1 mm. The angle deviation is obtained through geometric calculation and is related to the pipe diameter. When the outer diameter is 20 mm (i.e., the radius is 10 mm), at this time, the error is arctan (0.1/10) ≈ 0.57°, so the accuracy of the angle deviation is 0.57°.

**Figure 11:**
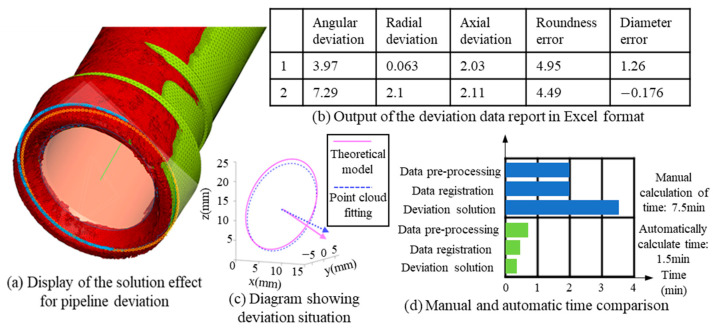
Schematic diagram of the deviation solution module. (**a**) Display of the solution effect for pipeline deviation; (**b**) Output of the deviation data report in Excel format; (**c**) Diagram showing deviation situation; (**d**) Manual and automatic time comparison.

To achieve the solution of the deviation, it is necessary to compare the normal vectors and end faces of the theoretical model with those of the measured point cloud data. The axis and end face of the theoretical model are known during the model definition. The normal vectors and end face information of the point cloud data are obtained by fitting after extracting the point cloud. By taking the axis as the normal vector to extract the end face to be measured, an ellipse is obtained through point cloud fitting. The solution of the deviation is shown in Figure 11a. The green part in the figure represents the theoretical model, the red part represents the point cloud data, the yellow circle extracted is the end face of the theoretical model, and the blue circle extracted is the end face of the point cloud data. Subsequently, a series of operations such as ellipse fitting are performed to obtain the end face information.

In addition, reports and screenshots are automatically generated to summarize and present the computational results and analysis conclusions. The report generation module utilizes data and charts from the computation and visualization modules to automatically produce comprehensive reports containing textual descriptions, graphical presentations, and data tables. This module supports exporting reports in Excel format, as illustrated in Figure 11b. It also retains a schematic diagram of the deviation conditions to visually convey the deviation status, as shown in Figure 11c. The comparison of the time required for manual solution and automatic solution is shown in Figure 11d. It can be seen that the operation time for single-root management can be shortened from 7.5 min to 1.5 min, saving 80% of the time for point cloud data processing and deviation solution, this is highly practical for the assembly process of the aircraft engine piping.

With the continuous development of AI technology, the state-of-the-art in industrial software development is widely regarded as the integration of intelligent manufacturing techniques and other AI technologies with traditional industrial software. Although the software developed in this paper has not fully integrated with AI technology, compared to the SOTA, although this software has poorer versatility, longer parameter adjustment cycles and requires reliance on data processing experience, it still has significant advantages in terms of final error solution accuracy and efficiency. Moreover, the operation process is simple and truly meets the needs of production line workers.

## 5. Conclusions

This study focuses on the non-contact measurement challenges inherent in aerospace engine pipeline systems by utilizing advanced 3D laser scanning technology to capture high-resolution point cloud data. To effectively process this data, a suite of dedicated point cloud processing algorithms was developed, including segmentation, noise filtering, and deviation analysis. These algorithms were integrated into an automated software platform designed for user-friendly, one-click operation, which facilitates rapid and accurate measurement of five critical pipeline deviation types: angular deviation, radial deviation, axial deviation, roundness error, and diameter error.

In addition to addressing the practical needs of industrial pipeline inspection, this work makes a significant methodological contribution by innovatively extending the traditional two-dimensional Douglas–Peucker (DP) algorithm into the three-dimensional domain. This 3D adaptation allows for efficient down sampling of point cloud data while preserving essential geometric and structural features, thereby enhancing computational efficiency without compromising measurement accuracy.

The software platform developed in this study not only automates the entire measurement workflow—from data import and preprocessing, through registration and feature extraction, to deviation reporting—but also supports detailed visualization and comprehensive report generation. This integrated approach significantly improves the efficiency and reliability of pipeline inspection in aerospace applications, providing a robust foundation for subsequent geometric modeling, assembly verification, and performance evaluation.

Overall, this research demonstrates the feasibility and effectiveness of combining advanced scanning technology, tailored point cloud processing algorithms, and automated software solutions to meet the stringent precision requirements of aerospace engine pipeline quality control.

## Figures and Tables

**Table 1:** Comparison Table of Common Point Cloud Segmentation Algorithms.

Method Type	Representative Algorithm	Applicable Scenarios	Advantages and Disadvantages
Geometric-driven segmentation	SDF	Structured scenarios	Fast speed but dependent on parameter adjustment
Deep learning voxel method	3D U-Net	Medical image segmentation	Resolution is limited, suitable for scenarios with uniformly distributed points
Deep learning point method	PointNet++	Autonomous driving	Geometric details are well preserved but the computational resource consumption is high
Graph convolution method	DGCNN	Complex topological structures	Can model domain relationships but the computational resource consumption is high.

## Data Availability

The original contributions presented in this study are included in the article. Further inquiries can be directed to the corresponding author.

## References

[1] Kim Y., Nguyen C.H.P., Choi Y. (2020). Automatic pipe and elbow recognition from three-dimensional point cloud model of industrial plant piping system using convolutional neural network-based primitive classification. Autom. Constr..

[2] Luo J., Ma L., Cui R., Ren Y. (2025). VWC-LSPF: Voxelized weighted centroid least squares plane fitting based on point cloud data. Opt. Laser Technol..

[3] Li X., Gan V.J.L., Li K., Li M. (2025). High-precision 3D BIM reconstruction for mechanical, electrical and plumbing components using terrestrial laser scanning and LiDAR point clouds. J. Build. Eng..

[4] Xi G., Wang C., Liu X., Xiao B., Ding Y. (2026). Point cloud denoising method based on local neighborhood features. Opt. Laser Technol..

[5] Chao J., Lei J., Zhou X., Xie L. (2025). A general and flexible point cloud simplification method based on feature fusion. Displays.

[6] Yang B., Ye X. (2025). Few-shot 3D point cloud segmentation with unknown class number. Knowl.-Based Syst..

[7] Wang N., Ma D., Du X., Li B., Di D., Pang G., Duan Y. (2024). An automatic defect classification and segmentation method on three-dimensional point clouds for sewer pipes. Tunn. Undergr. Space Technol..

[8] Ma J., Zhang Q., Chen W., Yan W., Shen J., Xie Y., Zeng X. (2025). A novel point cloud segmentation method for accurate surface partitioning based on feature boundaries in the machining of industrial components. Chin. J. Mech. Eng..

[9] Verhoeven V.B., Raumonen P., Åkerblom M. (2025). Fitting geometric shapes to fuzzy point cloud data. J. Imaging.

[10] Lipschütz H., Reitebuch U., Polthier K., Skrodzki M. (2026). Feature-aware manifold meshing and remeshing of point clouds and polyhedral surfaces with guaranteed smallest edge length. Comput.-Aided Des..

[11] Yan B., Tao Z., Lin S., Li H. (2025). Multi-level feature fusion parallel branching networks for point cloud learning. Comput. Graph..

[12] Zheng Q., Wu S., Wei J. (2025). VoxT-GNN: A 3D object detection approach from point cloud based on voxel-level transformer and graph neural network. Inf. Process. Manag..

[13] Zhang K., Zhang A., Wang X., Li W. (2024). Deep-learning-based point cloud completion methods: A review. Graph. Models.

[14] Tang X., Shao F., Mu B., Chen H. (2026). PU-MG: Mutual guidance framework for point cloud upsampling. Displays.

[15] Ding Z., Zhang G., Gao H.-A., Chen X., Fan Z., Ding N., Zhao H. (2025). Hoodie: Hierarchical point cloud and latent code diffusion for joint and conditional generation. Neurocomputing.

[16] Yang Y., Zhao Z., Yin F., Liu W., Ding Y., Jiang B., Yu G., Chen T. (2026). A unified multi-modality conditional latent diffusion model for point cloud generation. Pattern Recognit..

[17] Yu W., Shu J., Yang Z., Ding H., Zeng W., Bai Y. (2025). Deep learning-based pipe segmentation and geometric reconstruction from poorly scanned point clouds using BIM-driven data alignment. Autom. Constr..

[18] Wang S., Zhao W., Zhang H., Wei J., Zhang G. (2025). B-spline surface mapping for robust point cloud correlation and physical deformation measurement. Opt. Laser Technol..

[19] Si S., Zhang H., Guo J., Luo H., Ning X., Zhang Z., Yu Z., Mao N., Yu W. (2026). PointLMDA: Local masked reconstruction and structural prediction for self-supervised domain adaptation on 3D point clouds. Inf. Fusion.

[20] Song Y., He F., Fan L., Dai J., Guo Q. (2022). DSACNN: Dynamically local self-attention CNN for 3D point cloud analysis. Adv. Eng. Inform..

[21] Kumari S., Mandal S., Raman S. (2025). Structure preserving point cloud completion and classification with coarse-to-fine information. J. Vis. Commun. Image Represent..

[22] Sun L., Wang Y., Zhu Q. (2025). Dual-attention-based block matching for dynamic point cloud compression. J. Imaging.

[23] Tian D., Gong M., Li J., Shi J. (2026). 3D point cloud classification network with hybrid sampling enhancement and point energy attention. Pattern Recognit..

[24] Liang Y., An P., Liu Q., Yang Y., Xu L. (2026). Density-aware few-parametric networks for robust few-shot point cloud semantic segmentation. Neurocomputing.

[25] Zhao L., Hu Y., Yang X., Dou Z., Kang L. (2024). Robust multi-task learning network for complex LiDAR point cloud data preprocessing. Expert Syst. Appl..

[26] Deng H., Li S., Chen S., Liu C., Wang L. (2026). PointSEN: Understanding point cloud via shape-aware embedding and adaptive group normalization. Appl. Soft Comput..

[27] Xie T., Liu Q., Wang P., Chan R.H.M. (2025). Optimizing 3D point cloud representations for machine learning: Advances in down-sampling techniques. Neurocomputing.

[28] Li M., Feng X., Hu Q. (2023). 3D laser point cloud-based geometric digital twin for condition assessment of large diameter pipelines. Tunn. Undergr. Space Technol..

[29] Lu J., Wang Y., Wu Y., Li H., Shi Y., Ning F. (2026). An autoregressive framework for reconstructing editable parametric computer-aided design models from point clouds. Eng. Appl. Artif. Intell..

[30] Luo L., Lu J., Chen X., Zhang K., Zhou J. (2025). LGCANet:Local geometry-aware cross-attention networks for point cloud semantic segmentation. Measurement.

[31] Ding C., Liu Y., Cao T., Wang J. (2024). Aero-engine pipe gap automatic detection based on 3D scanning point clouds. Measurement.

[32] Zhang D., Yu L. (2026). Enhancing 3D point cloud generation via mamba-based time-varying denoising diffusion. J. Vis. Commun. Image Represent..

[33] Liu W., Gao F., Dong S., Wang X., Cao S., Wang W., Liu X. (2025). An enhanced segmentation method for 3D point cloud of tunnel support system using PointNet++ and coverage-voted strategy algorithms. J. Rock Mech. Geotech. Eng..

[34] Zhu S., Wang Y., Chen W., Wang Y. (2026). A progressive multilevel mixing-based knowledge distillation framework for enhancing three-dimensional object detection on compressed point clouds. Eng. Appl. Artif. Intell..

[35] Li F., Yan H., Xu X., Li Q. (2026). PU-mamba: A point cloud upsampling network to enhance semantic segmentation of MEP scenes using synthetic data. Adv. Eng. Inform..

[36] Feng Y., Feng S.-J., Zhang X.-L., Kong Q.-Z., Zhao Y. (2026). Automatic deformation detection of metro tunnels via point cloud segmentation and geometric analysis. Autom. Constr..

[37] Li C., Zhang P., Zhang J., Wu Y. (2026). Density-aware global–local attention network for point cloud segmentation. Image Vis. Comput..

[38] Zeng Y., Sun H., Li X., Chen Z. (2025). A multilevel feature-based method for mapping sparse point clouds to CAD models. Comput. Aided Geom. Des..

[39] Tang C., Wang H., Zhao J., Tang Y., Yan H., Xiao Y. (2021). A method for compressing AIS trajectory data based on the adaptive-threshold douglas-peucker algorithm. Ocean Eng..

[40] Wang Y., Cao J., Zheng X., Zhang Y., Zhang Y., Chen W. (2025). Path planning of RRT* algorithm with subregional dynamic probabilistic sampling based on artificial potential field in radiation environments. Nucl. Eng. Technol..

[41] Zygmunt M., Róg M. (2026). New approach towards digital elevation model data generalisation using the douglas-peucker algorithm and delaunay triangulation based on characteristic boundary points. Measurement.

